# Substances of emerging concern in Baltic Sea water: Review on methodological advances for the environmental assessment and proposal for future monitoring

**DOI:** 10.1007/s13280-021-01627-6

**Published:** 2021-10-12

**Authors:** Marion Kanwischer, Noomi Asker, Ann-Sofie Wernersson, Marisa A. Wirth, Kathrin Fisch, Elin Dahlgren, Helena Osterholz, Friederike Habedank, Michael Naumann, Jaakko Mannio, Detlef E. Schulz-Bull

**Affiliations:** 1 Department of Marine Chemistry, Leibniz Institute for Baltic Sea Research Warnemünde, Seestraße 15, 18119 Rostock, Germany; 2 Department of Biological and Environmental Sciences, University of Gothenburg, Medicinaregatan 18A, 41390 Göteborg, Sweden; 3 Department for Management of Contaminated Sites, Swedish Geotechnical Institute, Hugo Grauers gata 5 B, 41296 Göteborg, Sweden; 4 Swedish University of Agricultural Sciences, Stångholmsvägen 2, 178 93 Drottningholm, Sweden; 5 State Office for Agriculture, Food Safety and Fisheries, Mecklenburg-Western Pomerania, Thierfelderstraße 18, 18059 Rostock, Germany; 6 Centre for Sustainable Consumption and Production/Contaminants, Finnish Environment Institute, Latokartanonkaari 11, 00790 Helsinki, Finland; 7 Department of Physical Oceanography and Instrumentation, Leibniz Institute for Baltic Sea Research Warnemünde, Seestraße 15, 18119 Rostock, Germany

**Keywords:** Baltic Sea, Bioassay, Biomarker, Effect-based methods, Instrumental analysis, Substances of emerging concern

## Abstract

The Baltic Sea is among the most polluted seas worldwide. Anthropogenic contaminants are mainly introduced via riverine discharge and atmospheric deposition. Regional and international measures have successfully been employed to reduce concentrations of several legacy contaminants. However, current Baltic Sea monitoring programs do not address compounds of emerging concern. Hence, potentially harmful pharmaceuticals, UV filters, polar pesticides, estrogenic compounds, per- and polyfluoroalkyl substances, or naturally produced algal toxins are not taken into account during the assessment of the state of the Baltic Sea. Herein, we conducted literature searches based on systematic approaches and compiled reported data on these substances in Baltic Sea surface water and on methodological advances for sample processing and chemical as well as effect-based analysis of these analytically challenging marine pollutants. Finally, we provide recommendations for improvement of future contaminant and risk assessment in the Baltic Sea, which revolve around a combination of both chemical and effect-based analyses.

**Supplementary Information:**

The online version of this article (10.1007/s13280-021-01627-6) contains supplementary material, which is available to authorized users.

## Contaminant assessment in the Baltic Sea

Since the onset of industrialization at the end of the nineteenth century, large amounts of industrially derived chemicals have been released into the environment. After discovering harmful effects on ecosystems, identification and regulation of certain persistent contaminants began in the 1970s, about three to four decades after their first use. For example, the environmental concentration of dichlorodiphenyltrichloroethylene (DDE), the degradation product of the organochlorine pesticide dichlorodiphenyltrichloroethane (DDT) was found to be strongly linked to egg shell thinning in birds. This had severe consequences for several species on the population level. However, retroactive studies showed that such effects already occurred in the early 1950s (Peakall 1993). Not only ecosystems but humans as well have been affected by persistent contaminants, even far away from the emission sources. For example, in the 1980s it was discovered that Arctic Inuit populations had high concentrations of polychlorinated biphenyls (PCBs) in breast milk (Dewailly et al. 1989). While national measures to regulate use and emissions of some of the organic pollutants were implemented in the 1970s to 1980s, the global convention on persistent organic pollutants, the Stockholm Convention, was ratified in 2004 (for an overview see HELCOM 2010). Nevertheless, a range of legacy contaminants are still present in the environment at high concentrations, emphasizing the importance of early detection of potential substances of concern.

Marine environments are receptors of environmental contaminants, stemming from land-based activities, entering the sea through riverine input or airborne deposition, as well as sea-based activities. Depending on their physicochemical characteristics, water contaminants either remain in the water column or bind to particles that are eventually deposited at the seafloor. However, sediment contaminants can be re-mobilized and re-enter the water column, e.g., due to physical disturbance. Aquatic organisms are exposed to these contaminants from both water and sediment, but also indirectly, through the food chain.

**Fig. 1:**
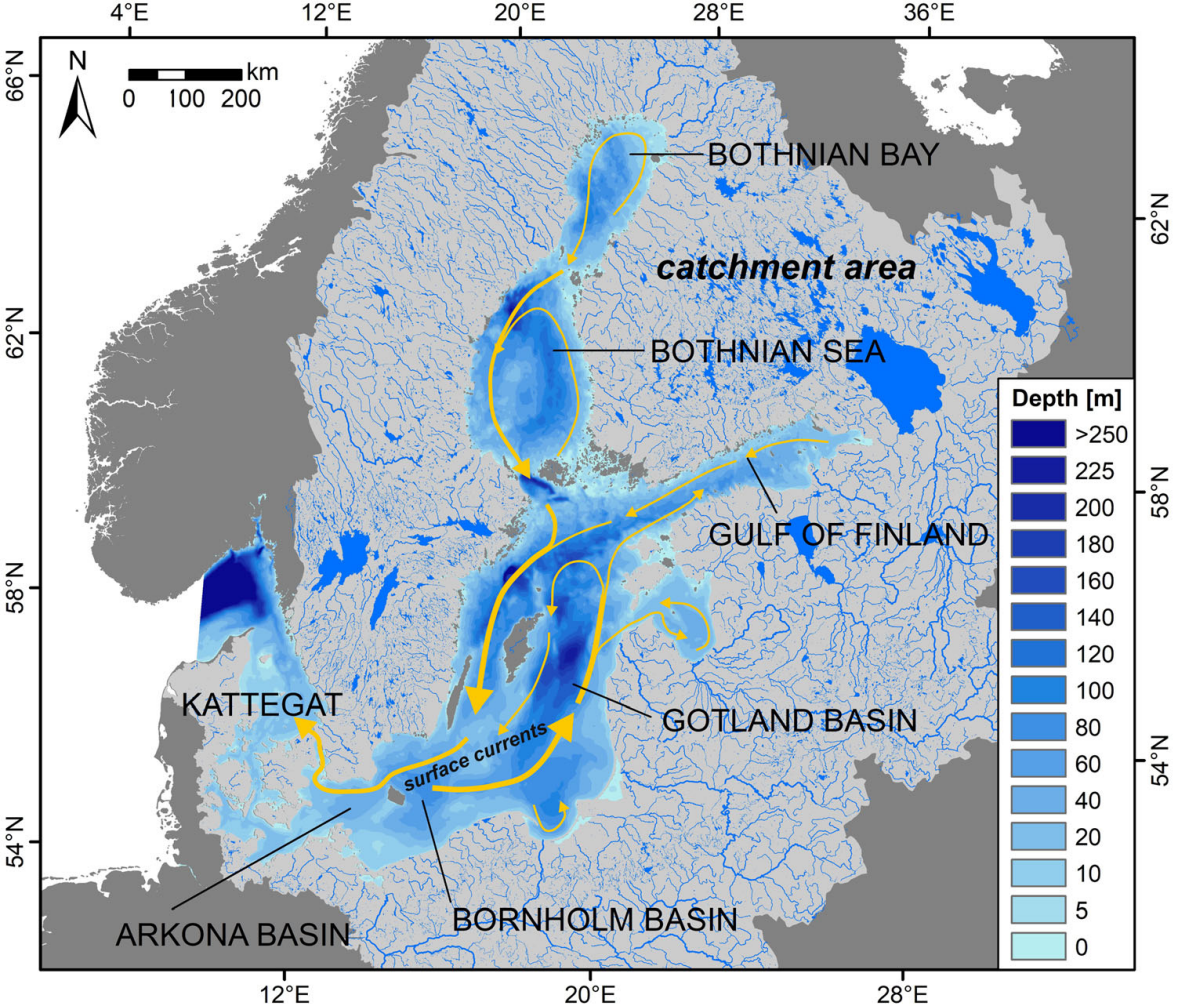
Map of the Baltic Sea and its catchment area. The Baltic Sea has a surface area of 412 560 km^2^, a water volume of 21 631 km^3^, and an average water depth of 52 m with a maximum of 460 m at the Landsort Deep. The sea floor topography structures the Baltic Sea into various sub-basins divided by sill areas. It is characterized by a permanently stratified water column with brackish water of low salinity in the surface water fed by riverine runoff and a deep water layer of higher salinity influenced by rare salt-water inflow events from the North Sea/North Atlantic. There is a strong salinity gradient from the entrance in the western part of the Baltic Sea to the central and north-eastern part with salinities close to freshwater conditions in the Bothnian Bay (Feistel et al. 2008). Catchment area (1 749 209 km^2^) is shown in light gray; rivers (in total 8478) are shown as blue lines, their widths correspond to the river length. The arrows show the main Baltic Sea surface water currents

The Baltic Sea is one of the most polluted seas in the world. It is a semi-enclosed sea with substantial riverine inflow with an annual average of about 14 000 m^3^/a (Johansson 2018). Its catchment area is about four times the size of the Baltic Sea itself, corresponding to about half of the size of Europe. Rivers carry large amounts of nutrients and hazardous contaminants into the Baltic Sea (Fig. 1). Besides this, there is also substantial airborne contaminant deposition of heavy metals and organic hazardous substances on the water surface (HELCOM 2018b).

Although the concentrations of several legacy pollutants are still exceeding different types of environmental quality standards, the measures have indeed led to decreasing concentration trends in the Baltic Sea in most cases (HELCOM 2018c,d), which is most evident in marine biota (Gustavsson 2010).

A revision proposal of the current monitoring system for the Baltic Sea including monitoring strategies for hazardous substances was the overall objective of the EU-financed BONUS SEAM project. It was concluded that current strategies in monitoring do not reflect compounds of emerging concern and it was recommended to increase efforts to determine relevant target substances and to appropriately address them in Baltic Sea monitoring (Kanwischer et al. 2019).

Analysis of seawater is most indicative of the contaminant sources and, thus, allows tracing their transport pathways into the Baltic Sea. However, there are only a few HELCOM water monitoring programs of hazardous substances in the Baltic Sea and the current programs generally address only a very limited number of the substances of emerging concern. Thus, seawater concentrations of, e.g., pharmaceuticals and UV filters are currently not easy to take into account in the assessment of the state of the Baltic Sea. Moreover, analyzing the very low concentrations typical for marine waters remains a constant challenge. Beside anthropogenically induced contamination, harmful cyanobacterial blooms in the Baltic Sea have increased in frequency, biomass, and duration in the last decades (e.g., Finni et al. 2001), but algal toxin concentrations are not regularly monitored. Finally, *effects* of emerging concern such as endocrine disruption are not addressed, as well.

Therefore, this study aims (i) to review reported Baltic Sea data for surface water concentrations on selected contaminants of emerging concern and in vitro bioassays measuring estrogenic effects as well as naturally produced algal toxins; (ii) to describe state-of-the-art methodological advances for the chemical analysis of these compounds in seawater and the use of effect-based methods; (iii) to provide recommendations for improved monitoring strategies to assess the state of the Baltic Sea also covering the selected contaminants of emerging concern.

## Approach

In this review, we focus on selected compound groups that are of emerging concern: pharmaceuticals, polar pesticides, estrogenic substances, per- and polyfluoroalkyl substances (PFAS), UV filters, and algal toxins (e.g., Diamond et al. 2011) (Table 1).

**Table 1:** Compound groups and chemical structures of example substances addressed in this review. Data for the octanol–water-partition coefficient (log *K*_OW_) and water solubility were obtained from the databases: ^a^Pubchem (https://pubchem.ncbi.nlm.nih.gov/ accessed 2021/01/21), ^b^European Chemicals Agency (https://echa.europa.eu/de/ accessed 2021/01/21), ^c^Toxin and Toxin Target Database (http://www.t3db.ca/toxins accessed 2021/01/25), ^d^DrugBank (https://go.drugbank.com accessed 2021/01/25). *Predicted data (not measured)

Compound group	Example substance	log *K*_OW_	Water solubility
Pharmaceuticals	Carbamazepine	2.45^a^	18 mg/L (25 °C)^a^
	Sulfamethoxazole (SMX)	0.89^a^	610 mg/L (37 °C)^a^
UV filters	2-Phenylbenzimidazole-5-sulfonic acid (PBSA)	2^a,*^	317 mg/L^d,*^
	Octocrylene	7.1^a,c^ 6.1^b^	9–153 µg/L (20 °C)^b^
Estrogens	Ethinylestradiol (EE2)	3.67^a^	11.3 mg/L (27 °C)^a^
	Estrone (E1)	3.13^a^	0.03 mg/L (25 °C)^a^
Polar pesticides	Atrazine	2.61^a^	33 mg/L (25 °C)^a^
	Simazine	2.18^a^	6.2 mg/L (pH 7, 20 °C)^a^
Per- and polyfluoroalkyl substances	Perfluorooctane sulfonic acid (PFOS)	4.49^a^	3.2 µg/L (25 °C)^a^
Algal toxins	Nodularin	1.7^a,c^	7 mg/L^c,*^

A literature search was conducted to collect data on these substance groups in Baltic Sea surface water. The literature was also reviewed for state-of-the-art methods and recently reported advances in the field of sample processing, analysis approaches, and effect-based methods relevant for the determination, identification, and assessment of the substances addressed herein with particular focus on marine water. The searches were conducted, basically, following the process described by Mengist et al. (2020) and the details of the searches are summarized in Table S1.

## Anthropogenic and naturally derived substances of emerging concern in the Baltic Sea

### Current inclusion in water policy

Monitoring of organic hazardous substances in the Baltic Sea is predominantly conducted under the policies of the HELCOM commitment within the scope of the Baltic Sea Action Plan and EU legislation. Within the Water Framework Directive (WFD) context, priority substances listed in the Environmental Quality Standards Directive (EQSD) need to be monitored in water bodies they are emitted into. Monitoring data are used in the chemical status assessment. Substances included in the Watch list, established according to the EQSD, are also to be monitored, but during a shorter time period and on a limited number of sites. When sufficient monitoring data are available to draw conclusions about whether or not the substance could be of EU-wide concern and therefore should be considered for inclusion in the EQSD, the substance is removed from this list. In addition, each member state has an individual list of WFD river basin specific pollutants (RBSP) and the results are taken into account in the ecological status classification. Within the Marine Strategy Framework Directive (MSFD), both priority substances and RBSPs of relevance to the marine environment are taken into account in the assessment of the environmental status. Individual member states have also implemented additional MSFD indicators such as effect-based methods. Assessment under HELCOM relies on the indicator concept. Core indicators are agreed along with quantitative threshold values, whereas pre-core indicators have not reached the core indicator status yet and data do not enter the holistic assessment. The assessment is mostly based on chemical analytical data. So far, only the mandatory effect-based method *imposex* is included in the HELCOM monitoring program, which is very specific to tributyltin and cannot be expected to respond to any of the substances of emerging concern in focus of this review. Table 2 provides an overview of how the substances in focus of this review are currently addressed in HELCOM and the MSFD/WFD context.

**Table 2:** Selected substances for this review and their current inclusion in MSFD/WFD and HELCOM assessments. For a current and full list of HELCOM core indicators, see https://helcom.fi/baltic-sea-trends/indicators/ (^a^removed from the updated watch list; n.a. not addressed)

Compound group	WFD/MSFD		HELCOM	
	Substance	Inclusion	Indicator/index	Inclusion
Pharmaceuticals	Diclofenac^a^ Macrolide antibiotics Amoxicillin Ciprofloxacin	Current or previous watch list of the EQSD	Diclofenac	Pre-core test indicator 1° Matrix: seawater 2° Matrix: biota
UV filters	2-Ethylhexyl-4-methoxycinnamat^a^	Current or previous watch list of the EQSD	n.a.	n.a.
Estrogens	Estrone^a^ 17α-Ethinyl estradiol^a^ 17β-Estradiol^a^	Current or previous watch list of the EQSD	n.a.	n.a.
Polar pesticides	Simazine Atrazine Diuron Isoproturon Terbutryn	Priority substances of EQSD	n.a.	n.a.
	Methiocarb^a^ Neonicotinoids^a^	Current or previous watch list of the EQSD	n.a.	n.a.
Per- and polyfluoroalkyl substances	PFOS	Priority substance of EQSD	PFOS	Core indicator 1° Matrix: biota 2° Matrix: seawater
Algal toxins	n.a.	n.a.	Cyanobacterial bloom index	Pre-core indicator

### Reported data for Baltic Sea surface water

The herein addressed compounds are generally analyzed in a campaign-wise manner, such as screening studies, or within research contexts. In the following, they are shortly introduced and accessible data published for Baltic Sea surface water are summarized in Table 3.

**Table 3:** Reported data for Baltic Sea surface water on substances from the group of pharmaceuticals, UV filters, estrogens/estrogenic activity, PFAS, polar pesticides, and algal toxins. Data are listed as single concentrations, concentration ranges, or maximum analyzed concentration. *CMD, Chloridazon-methyl-desphenyl; 2,4-D, 2,4-Dichlorophenoxy-acetic acid; FOSA, Perfluoroctylsulfonamide; FOSAA, 2-(Perfluorooctanesulfonamido)acetic acid; 6:2 FTSA, 6:2 Fluorotelomer sulfonate; HFPO-DA, 2,3,3,3-Tetrafluoro-2-(1,1,2,2,3,3,3- 53 heptafluoropropoxy)propanoic acid; MCPA, 4-Chloro-2-methyl-phenoxy acetic acid; PFBA, Perfluorobutanoic acid; PFBS, Perfluorobutanesulfonic acid; PFDA, Perfluorodecanoic acid; PFDoDA, Perfluorododecanoic acid; PFECHS, Potassium perfluoro-4-exthylcyclohexanesulfonate; PFHpA, Perfluoroheptanoic acid; PFHxA, Perfluorohexanoic acid; PFHxS, Perfluorohexanesulfonic acid; PFNA, Perfluorononanoic acid; PFPA, Perfluorophosphonic acid; PFPeA, Perfluoropentanoic acid; PFTeDA, Perfluorotetradecanoic acid; PFUnDA, Perfluoroundecanoic acid; SPE, Solid phase extraction; LC, Liquid chromatography; ESI, Electrospray ionization ; MS, Mass spectrometry*

Compound class	Analysis method	Method details	Concentrations of analyzed substances (ng/L)	Baltic Sea area	Year	References
Pharmaceuticals	Chemical	SPE, LC–ESI–MSMS	Atenolol: ≤ 13, carbamazepine: ≤ 157, cetirizine: ≤ 13, clarithromycin: ≤ 14, diclofenac: ≤ 9.2, ibuprofen: ≤ 109, iohexol: ≤ 861, iomeprol: ≤ 1159, iopamidol: ≤ 1027, iopromide: ≤ 109, loratadine: ≤ 4.1, metoprolol: ≤ 158, paracetamol: ≤ 48, phenazone: ≤ 5.9, roxithromycin: ≤ 16, sotalol: ≤ 65, SMX: ≤ 42	German Baltic Sea coastline	2009	Nödler et al. (2014)
		SPE, LC–ESI–MSMS	Trimethoprim: 0.6, sulfadimethoxine: 0.7	Gulf of Gdansk	2011, 2012	Borecka et al. (2013)
		SPE, LC–ESI–MSMS	Trimethoprim: 1.4–2.2, SMX: 5.4–18.0, sulfadimethoxine: 0.5–1.0	Gulf of Gdansk	2012	Borecka et al. (2015)
			Trimethoprim: ≤ 1.6	Pomeranian Bight		
			Trimethoprim: 2.8	Gdansk Deep		
		SPE, LC–ESI–MSMS, LC–ESI-Qtrap	Carbamazepine: 0.5–12.2, clofibric acid: < 0.4, diclofenac: < 27.1, oxazepam: < 1.8, primidone: 1.1–5.8, metoprolol: 0.09–0.8	Baltic Sea	2001–2014	Fisch et al. (2021)
		Information not provided	Diclofenac: ≤ 54, ibuprofen: ≤ 158, naproxen: ≤ 14, phenazone: ≤ 504, paracetamol: ≤ 36, tramadol: ≤ 1.6, erythromycin, clarithromycin, azithromycin: ≤ 0.27, SMX: ≤ 33, metaprolol: ≤ 55, bisoprolol: ≤ 128, sotalol: ≤ 24, carbamazepine: ≤ 73, oxazepam: ≤ 1.9, primidone: ≤ 5.8, salicylic acid: ≤ 14, 17β-estradiol: ≤ 1.1, clofibric acid: ≤ 0.4	Samples from entire Baltic Sea area	2003–2014	UNESCO and HELCOM (2017)
		SPE, LC–ESI–MSMS	SMX: ca.1.5, salicylic acid: ca. 11	German Baltic Sea coastline	2015	Fisch et al. (2017)
UV filters	Chemical	SPE, LC–ESI–MSMS	PBSA: ≤ 3.4	Baltic Sea	2014	Orlikowska et al. (2015)
			PBSA: ≤ 170, BP-1: ≤ 2.5, BP-4: ≤ 226	German Baltic Sea coastline		
			PBSA: ca. 2–10, octocrylene: ca. 8–31	German Baltic Sea coastline	2015	Fisch et al. (2017)
Estrogens and estrogenic compounds	Effect-based	SPE, YES	EEQ: 0.22	Darss Peninsula	2003	Beck et al. (2006a)
			EEQ: 0.31	Salzhaff		
	Chemical	SPE, LC–ESI–MSMS	E1: 0.16–0.33, EE2: ≤ 2.1	Outer Wismar Bay	2003–2004	Beck et al. (2005)
			E1: 0.10–0.25, EE2: 1.7–2.5	Darss Peninsula		
			E1: 0.27–0.34, EE2: 1.7–2.9	Salzhaff		
	Effect-based	SPE, A-YES	EEQ: ≤ 0.11	Western Baltic Sea	2016–2018	Deich et al. (2020)
			EEQ: ≤ 0.38	German coastal Baltic Sea		
Polar pesticides and metabolites	Chemical	SPE, LC–ESI–MSMS	Atrazine: ≤ 2.1, desethylatrazine: ≤ 2.0, terbuthylazine: ≤ 7.2	German Baltic Sea coastline	2009–2010	Nödler et al. (2013)
			Desethylatrazine: ≤ 2.2, diuron: ≤ 131, isoproturon: ≤ 7.2, mecoprop: ≤ 18	German Baltic Sea coastline	2009	Nödler et al. (2014)
			Atrazine: ≤ 2.6, simazine: ≤ 3.5, terbuthylazine: ≤ 3.8, chloridazon: ≤ 7.4, CMD: ≤ 8.9, chlorotoluron: ≤ 2.7, diuron: 2.9, isoproturon: 6.6, bentazone: 1.1, 2,4–D: ≤ 3.2, metazachlor: ≤ 2.5	Baltic Sea	2014	Orlikowska et al. (2015)
			Atrazine: ≤ 7.6, simazine: ≤ 5.8, terbuthylazine: ≤ 1111, terbutryn: ≤ 10.5, irgarol: ≤ 1.9, desisopropylatrazine: ≤ 4.6, desethylathrazine: ≤ 2.5, chloridazon: ≤ 126, CMD: ≤ 32.9, chlorotoluron: ≤ 136, diuron: 107, isoproturon: 60.7, bentazone: 221, 2,4–D: ≤ 19.6, MCPA: ≤ 36.3, mecoprop: ≤ 9.7, metazachlor: ≤ 27	German Baltic Sea coastline		
			CMD: 1.4–8.9, isoproturon: ≤ 7.2, chloridazon: 2.1–6.9, bentazon: ≤ 1.1	German coastal Baltic Sea	2012–2104	Skeff et al. (2017)
		SPE, LC–ESI–MSMS, LC–ESI-Qtrap	Chlorotoluron: 0.03–6.8, diuron: 0.3–20.2, fenuron: ≤ 0.9, isoproturon: 0.04–17.8, linuron: ≤ 0.4, monolinuron: ≤ 0.04, 2,4-D: 0.2–85.3, dichlorprop: ≤ 2.6, MCPA: 0.1–2.6, mecoprop: ≤ 2.0, malathion: ≤ 0.3, ametryn: ≤ 0.1, atrazine: 1.0–26.0, desethylatrazine: 0.6–1.8, diazinon: ≤ 0.2, hexazinone: 0.04–0.4, irgarol: ≤ 4.9, prometryn: 0.1–0.9, propazine: 0.08–0.3, simazine: 0.9–4.3, terbuthylazine: 0.2–5.2, terbutryn: 0.01–0.6, chloridazone: 1.4–5.3, metazachlor: 0.01–8.9, methabenzthiazuron: 0.01–0.1, metolachlor: ≤ 1.5, pendimethalin: ≤ 1.1	Baltic Sea	2001–2014	Fisch et al. (2021)
		SPE, LC–ESI–MSMS	glyphosate: ≤ 1.22, aminomethylphosphonic acid: ≤ 1.42	Baltic Sea	2019	Wirth et al. (2021)
PFAS	Chemical	SPE, LC–ESI–MSMS	PFOA: 0.47–0.89, PFOS: 0.33–0.58	Western Baltic Sea	2004–2005	Theobald et al. (2011)
			PFOA: 1.1, PFOS: 0.9	Pomeranian Bight		
		SPE, LC–ESI–MSMS	PFBA: ≤ 0.44, PFPA: ≤ 0.12, PFHxA: 0.12–0.27, PFHpA: 0.06–0.26, PFOA: 0.25–4.55, PFNA: 0.10–0.42, PFBS: 0.26–0.88, PFHxS: ≤ 0.61, PFOS: ≤ 0.35, FOSA: ≤ 0.46	Baltic Sea	2007	Ahrens et al. (2010)
		SPE, LC–ESI–MSMS	PFBS: ≤ 0.76, PFHxS: ≤ 0.23, PFOS: 0.04–0.39, PFPA: ≤ 0.18, PFHxA: 0.09–0.29, PFHpA: ≤ 0.2, PFOA: 0.12–0.78, PFNA: 0.09–0.85, PFDA: ≤ 0.15, PFDoDA: ≤ 0.3	Baltic Sea	2008	Kirchgeorg et al. (2010)
		SPE, LC–ESI–MSMS	PFBA: 0.34–0.67, PFHpA: 0.61–1.0, PFOA: 0.21–1.3, PFNA: 0.14–5.7, PFDA: 0.045–0.83, PFDoDA: 0.045, PFTeDA: 0.016–0.072, PFBS: 0.062–0.57, PFHxS: 0.11–1.7, PFOS: 0.11–2.5, FOSAA: 0.061, FOSA: 0.019–0.051	Baltic Sea	2013	Nguyen et al. (2017)
		SPE, LC–ESI–MSMS, LC–ESI-Qtrap	PFBS: < 0.2, PFHxA: 0.04–0.4, PFHpA: 0.1–0.4, PFHxS: 0.06–0.3, PFNA: 0.07–0.3, PFOA: 0.3–1.0, PFOS: 0.1–0.8, PFDoDA: < 0.07, FOSA: < 0.007	Baltic Sea	2001–2014	Fisch et al. (2021)
		SPE, LC–ESI–MSMS	HFPO-DA: ≤ 0.082, PFECHS: ≤ 0.14, PFBA: 0.33–0.99, PFPeA: ≤ 0.75, PFHxA: 0.22–0.84, PFHpA: ≤ 0.38, PFOA: 0.20–0.70, PFNA: ≤ 0.21, PFDA: ≤ 0.047, PFBS: ≤ 0.43, PFHxS: ≤ 0.48, L-PFOS: ≤ 0.082, Br-PFOS: 0.029–0.098, 6:2 FTSA: ≤ 0.93, L-FOSA: ≤ 0.0064, Br-FOSA: ≤ 0.0064	German Baltic Sea coastline	2017	Joerss et al., 2019
Algal toxins	Chemical	SPE, LC–UV	Extracellular nodularin: 90–18 000 µg/L	Gulf of Gdansk	2001–2002	Mazur and Plinski (2003)

The presence of **pharmaceuticals** and their transformation products in the marine environment has received large attention in recent years due to observed harmful effects on non-target species after environmental exposure (e.g., Alygizakis et al. 2016; reviewed by Białk-Bielińska et al. 2016; Ojemaye and Petrik 2019). They enter the marine environment indirectly with treated and untreated waste water from households, agriculture, or industry (Heberer and Ternes 2006; Gaw et al. 2014). Their ecotoxicity presumably derives from their mode of action. Gunnarsson et al. (2008) showed that there are drug targets conserved among humans and aquatic species, which is a basis for potential interaction of pharmaceuticals with wildlife when released into the environment.

Common pharmaceutical substances analyzed in Baltic Sea surface water derive from the therapeutic groups of anti-inflammatory and analgesic agents, cardiovascular and central nervous system agents, antimicrobials, X-ray contrast media, and antiallergic agents. The compounds diclofenac, ibuprofen, paracetamol and phenazone, metoprolol, carbamazepine (Nödler et al. 2014; UNESCO and HELCOM 2017), sulfamethoxazole (SMX; Borecka et al. 2013, 2015; Nödler et al. 2014; Fisch et al. 2017; UNESCO and HELCOM 2017) as well as iopamidol and iomeprol (Nödler et al. 2014; Kötke et al. 2019) were the most frequently measured substances. In addition, Björlenius et al. (2018) conducted a Baltic Sea wide study on 93 pharmaceutical substances and detected 39 of them in surface water samples. Carbamazepine was the most frequently detected substance in that study.

**UV filters** are frequently included in personal care products such as sunscreen formulations or polymer-based products. The main source of UV filters into the marine environment is directly via recreational activities (Díaz‐Cruz and Barceló 2015). They are under ongoing investigation for being potentially persistent, bioaccumulative, and toxic in the environment (reviewed by Brausch and Rand 2011; Sánchez-Quiles and Tovar-Sánchez 2015). Endocrine disruptive effects were shown for the UV filter oxybenzone (Schlenk et al. 2005; Zwart et al. 2018). Due to their contribution to coral bleaching, the distribution of sunscreen formulations containing oxybenzone or octinoxate is banned in Hawaii to preserve the marine ecosystem (Hawaii Senate Bill 2571). Even though UV filters enter the environment mostly through the water phase, they can accumulate in different compartments such as sediments and biota (Sánchez-Quiles and Tovar-Sánchez 2015).

While UV filters were shown to be widely present in the marine environment (e.g., Rainieri et al. 2017), only few reports exist for the Baltic Sea (Orlikowska et al. 2015; Fisch et al. 2017; Apel et al. 2018). In Baltic Sea surface water, the UV filters 2-phenylbenzimidazole-5-sulfonic acid (PBSA), octocrylene, benzophenone (BP)-1, and BP-4 were detected, with PBSA and octocrylene as the most frequently detected compounds (Orlikowska et al. 2015; Fisch et al. 2017).

**Estrogens and estrogenic compounds** and their endocrine disruptive effects on organisms are largely in current focus of environmental and marine science (Hotchkiss et al. 2008; Arditsoglou and Voutsa 2012; Cotrim et al. 2016). The majority of the estrogenic activity in the environment presumably derives from naturally occurring and synthetic estrogens such as estrone (E1), 17β-estradiol (E2), the anthropogenic 17α-ethinyl estradiol (EE2), and estriol (Jarošová et al. 2014). This subject is often addressed through chemical analysis of estrogenic compounds, but also through the assessment of effects that are specific to estrogenic pressure and expressed as estradiol equivalent concentrations (EEQ). In the Baltic Sea, studies were mainly conducted at coastal sites and estrogen concentrations as well as estrogenic effects were determined (Beck et al. 2005, 2006a,b; Deich et al. 2020). Overall, E1 and EE2 are the predominant estrogens in Baltic Sea surface water (Beck et al. 2005, 2006b).

Widespread and large application of **pesticides** in industrial cropping and agriculture, domestic use, and other fields of application is of environmental concern due to the adverse effects they might have on non-target species. Compounds in current focus of environmental research belong to the group of polar pesticides such as triazines (e.g., atrazine and simazine), phenoxyacid herbicides [e.g., mecoprop and 2,4-dichlorophenoxy-acetic acid (2,4-D)], urea herbicides (e.g., diuron and chlorotoluron), or neonicotinoids (e.g., imidacloprid). Data on the occurrence of polar pesticides in surface water of the Baltic Sea and its coast in recent years were reported by Nödler et al. (2013, Nödler et al. 2014), Orlikowska et al. (2015), Skeff et al. (2017), and Fisch et al. (2021) and the most frequently addressed substances were diuron and isoproturon.

**Per- and polyfluoroalkyl substances** (PFAS) are utilized for technical applications such as fire extinguishing agents and the production of water and oil-repellent coatings. PFAS and their metabolites are known for their toxicity towards biota (DeWitt 2015). The PFAS compounds perfluorooctane sulfonate (PFOS) and perfluorooctanoic acid (PFOA) are internationally restricted under the Stockholm Convention. However, regulation of this compound class is exceptionally challenging due to the huge number of individual substances and new, structurally similar PFAS constantly entering the market. Therefore, the degree of environmental contamination by this compound class is currently not fully understood (Wang et al. 2017).

A mass balance performed by Filipovic et al. (2013) for selected PFAS compounds showed that riverine inflow and atmospheric deposition are predominant sources of PFAS to the Baltic Sea and a range of long-chain and short-chain PFAS were detected in Baltic Sea surface water (Ahrens et al. 2010; Kirchgeorg et al. 2010; Theobald et al. 2011; Nguyen et al. 2017; Joerss et al. 2019; Fisch et al. 2021). However, Johansson and Underman (2020) pointed out that based on studies applying methods to detect total extractable fluorine, the so far known PFAS compounds probably represent only 10–40% of total PFAS in environmental matrices.

**Naturally produced algal toxins** are of concern for the Baltic Sea due to the increased frequency, biomass and duration of harmful blooms of cyanobacteria and filamentous algae (e.g., Finni et al. 2001). The HELCOM holistic assessment from 2018 showed that the cyanobacterial bloom index failed in all assessed areas (HELCOM 2018a). The presence of cyanotoxins such as the hepatotoxin nodularin, polybrominated phenols such as tribromophenols, hydroxyl- and methoxylated polybrominated diphenyl ethers (OH-and MeO-PBDEs), and polybrominated dibenzodioxins has been confirmed in all compartments of the Baltic Sea food web, from primary producers such as algae and bacteria (Malmvärn et al. 2008), to mussels, fish (Sipiä et al. 2007; Löfstrand et al. 2011), seals (Routti et al. 2009; Lindqvist and Asplund 2019), and birds (Sipiä et al. 2008; Nordlöf et al. 2012). Metabolic transformation processes of the PBDEs ultimately lead to the accumulation of the most toxic congener 6-OH-BDE47 at the top of the food chain (Dahlgren et al. 2016). Concentration fluctuations by three orders of magnitude within a few weeks have been observed in blue mussel (*Mytilus edulis*) (Löfstrand et al. 2011) and the filamentous algae *Ceramium tenuicorne* (Dahlgren et al. 2016). In Baltic Sea studies, extracellular and cell-bound nodularin analysis was conducted in seawater following blooms of the cyanobacterium *Nodularia spumigena*, showing large variations in nodularin concentration in the water column along with a high turnover rate (Mazur and Plinski 2003; Carlsson and Rita 2019). These properties render the naturally produced toxins complicated to fit into the framework of national monitoring programs, for which sampling are often conducted in longer intervals.

## Advances and state-of-the-art methods for chemical analysis of the selected compound groups in seawater

### Methods for seawater sample processing

Compared to many of the legacy contaminants, several of the herein addressed substances are characterized by lower log *K*_OW_ values, which imply a higher polarity of these substances. Hence, many of them possess substantial water solubility (Table 1), which can be the main obstacle in sample preparation and instrumental analysis. The comparably low concentrations in the marine environment usually require **sample enrichment** during sample preparation.

Large-volume injection of up to 5 mL was established in LC–MS(MS) analysis as a way to overcome low concentrations in environmental water samples and to replace pre-enrichment steps, e.g., during the analysis of pesticides and pharmaceuticals (reviewed by Busetti et al. 2012). Dispersive liquid–liquid microextraction was used for the pre-concentration of samples for the analysis of UV filters (Benedé et al. 2014) or PFAS (Concha-Graña et al. 2018) in seawater. However, if higher enrichment factors are required, solid phase extraction (SPE) is the method of choice for UV filters (Bratkovics and Sapozhnikova 2011), pharmaceuticals (Paíga et al. 2015; Białk-Bielińska et al. 2016), pesticides (Loos et al. 2013; Rodríguez-González et al. 2017; Li et al. 2019; Xiao et al. 2021), estrogens (Rocha et al. 2012; Ronan and McHugh 2013; Heub et al. 2015), and PFAS (Loos et al. 2013; Brumovský et al. 2018). Besides classical off-line SPE approaches, automated procedures directly coupled to LC systems have been utilized in the last years, e.g., for the analysis of UV filters in seawater samples (Oliveira et al. 2010; Montesdeoca-Esponda et al. 2012), triazine herbicides (Rodríguez-González et al. 2016), and algal toxins (Zhang et al. 2018; Merlo et al. 2020; Wang et al. 2021).

Recently, a group of resins have become available which base on the target analytes’ specific molecular recognition sites and, therefore, allow a highly selective sample extraction and sample enrichment (Ansari and Karimi 2017). Those tailor-made molecularly imprinted polymers were, for example, successfully utilized for the analysis of the antibiotic sulfadiazine (Lian et al. 2014) and the herbicide glyphosate in Baltic Sea water (Wirth et al. 2021).

In view of the water solubility of the target analytes, finding a suitable SPE material can be challenging, particularly, if the target analyte has ionic characteristics. In this case, the application to seawater is largely restricted, as sea salt will bind to the binding sites of the SPE resin, avoiding efficient retention of the target analyte onto the column. In this regard, it has recently been shown that **electrodialysis** (ED) is a promising sample processing tool for the targeted analysis of pollutants from seawater (Wirth et al. 2019, 2021; Lohrer et al. 2020). ED is established in many industrial processes, e.g., for seawater desalination during drinking water production, but has found limited application in environmental sciences, so far. ED can be used to reduce the sample salinity prior to further sample processing and analysis. Improving effects such as enhanced sensitivity during mass spectrometric (MS) analysis could be shown (Wirth et al. 2019). However, analytes can be lost from samples via ED membrane passage or system wall adsorption. In a detailed investigation on the recovery of a wide polarity range of target pollutants, compounds of medium polarity (log *K*_OW_ − 1 to 3) were found to have especially high recoveries after the desalination process. Furthermore, compounds of high polarity (log *K*_OW_ <  − 1), e.g., the herbicide glyphosate, were found to only have decreased recoveries at low residual salinities. Thus, such compounds are also suitable for ED-based sample preparation, as long as ED is terminated before significant loss occurs and the subsequent analytical procedure tolerates the residual salt content.

### Analytical approaches

**Chromatographic separation** through LC is analytically very versatile and it is currently the predominant separation technique for the herein addressed substances due to their hydrophilic properties (Wille et al. 2012; Noguera-Oviedo and Aga 2016). However, with LC–-MS(MS)-based methods, matrix effects may emerge and should be carefully evaluated, in particular, if ionization is conducted with electrospray ionization (ESI; Busetti et al. 2012; Magi and Di Carro 2018).

**MS methods** are still the method of choice for qualitative and quantitative analysis (Magi and Di Carro 2018). Within the last decades, MS sensitivity was enhanced considerably: Detection levels for triple quadrupole tandem mass spectrometers (MSMS) have been reduced to sub-femtogram on column level, and simultaneous increase in scan rates allows analysis of an increased number of target compounds; hence multi-class methods with up to 1000 multiple-reaction monitoring (MRM) transitions within a single chromatography run are not uncommon anymore (Wille et al. 2012; Sulyok et al. 2020). Compared to single quadrupole technology, routine application of MRM transitions per analyte has enhanced selectivity and identification via the detection of a number of fragment ions next to the quasi-molecule ion. However, tandem mass spectrometry with preselected MRM transitions reduces the analysis to preselected targets.

High-resolution mass spectrometry (HR-MS), e.g., time-of-flight (TOF) or Orbitrap analyzers allow the determination of exact mass combined with high resolving power. Increased resolving power enables the analysis of lower analyte concentrations in more complex matrices with enhanced mass selectivity (Leendert et al. 2015). To date, TOF mass analyzers attain mass resolving power of 50 000 or higher, while Orbitrap systems can reach a resolving power above 1 000 000 (e.g., Schmidt et al. 2018). Additional fragmentation (MS^n^) makes both analyzers more selective than accurate-mass detectors alone (Zubarev and Makarov 2013). Hence, today, most TOF and Orbitrap analyzers are employed in hybrid with a quadrupole or ion trap, rather than in stand-alone configuration (Maurer and Meyer 2016).

**High-resolution continuum molecular absorption spectrometry** was used for the complex field of fluorinated organic compounds, which also includes the group of PFAS (Metzger et al. 2019). Instead of the typical target analysis, this approach yields a quantitative value for the sum of extractable organically bound fluorine in an environmental sample, which mostly has anthropogenic origin. In combination with SPE, the developed method was utilized for a screening in riverine surface water with detection limits in the low ng/L range. Akhdhar et al. (2020) showed that this approach might also be applicable to marine surface water analysis.

**Non-targeted and suspect analysis** approaches are gaining increasing interest, as known chemicals comprise only a small proportion of the contaminant mixture in the environment. Also in water policy this approach is considered supportive for the detection and identification of emerging contaminants (Leendert et al. 2015; Hollender et al. 2019). Fourier-transform ion cyclotron resonance mass spectrometry and Orbitrap instruments, but also untargeted MSMS have been applied across terrestrial and marine environments to decipher the biogeochemical cycling of carbon compounds (e.g., Longnecker and Kujawinski 2017). These analytical approaches equally enable the study of the anthropogenic chemical load in the environment. TOF-MS in combination with a molecular feature extraction algorithm was used in suspect analysis for the identification of the so far unknown PFAS compounds in river water (Strynar et al. 2015) while a hybrid Orbitrap-HRMS was used for seawater (Concha-Graña et al. 2018). Thus, for the identification of unknown PFAS, in particular, HRMS has become indispensable (Gao et al. 2020). A combination of untargeted and targeted screening of the sample to detect new emerging contaminants and their transformation products can also provide security in production processes (Hogenboom et al. 2009). In the marine environment, untargeted screening revealed the presence of pharmaceuticals, personal care products, and pesticides via LC-Orbitrap-HRMS in the Belgian North Sea in trace amounts (Vanryckeghem et al. 2019). Using untargeted HRMS, Lara-Martín et al. (2020) identified more than 500 sewage-derived contaminants in the NE Atlantic and could trace the anthropogenic imprint beyond the continental margin. Even in the sensitive Antarctic ecosystem, the analysis of algal metabolomes via FT-ICR-MS revealed the presence of several persistent and pharmaceutical compounds (Duarte et al. 2021).

The complex matrix of seawater might hamper the detection of contaminants due to the high risk of false-positive signals from compounds of the same exact mass. Currently, only the combination of different analytical approaches such as HR-MS, MSMS, and NMR can provide the necessary level of confirmation (Agüera et al. 2013; Ruan and Jiang 2017), but the unambiguous identification of compounds remains difficult due to the limitation of spectral and structure library coverage. Schymanski et al. (2014) communicated a level system on identification confidence in HR-MS analysis, which is widely followed in the community. Online databases or tools such as massbank or GNPS (Horai et al. 2010; Wang et al. 2016) are growing and allow for meta- or re-analysis of datasets as the database entries increase.

### Passive (integrative) sampling

The free dissolved concentration of a compound is important for the evaluation of its bioavailability to predict toxic effects and accumulation in the food web. The fraction of the free dissolved concentration of trace substances in water can only be determined with passive sampling. During passive sampling, trace substances adsorb on suitable materials and are concentrated onto the sampler from the surrounding medium. Extraction of the adsorbed substances from the sampler and their target or non-target/suspect analysis is conducted according to their chemical characteristics and the overall aim of the integrative sampling.

Adsorption of contaminants onto the sampler is time dependent and, thus, passive sampling provides integrated results over a period of exposure time (Smedes and Booij 2012). Physicochemical principles of passive sampling have been investigated in detail and evaluated in field and laboratory studies (Booij et al. 2007; Smedes et al. 2009; Morin et al. 2018). Required exposure times to collect sufficient amount of the substance primarily depend on the target substance and range from several days for substances with log *K*_OW_ of approx. 4 up to years with log *K*_OW_ > 7 (Booij et al. 2016). However, exposure to a sampler is not unlimited due to disturbing effects such as biofouling or changing environmental conditions.

Passive sampling devices have been developed for a range of applications in seawater (reviewed by e.g., Namieśnik et al. 2005; Vrana et al. 2005; Taylor et al. 2019), however, mostly for non-polar organic contaminants (e.g., Lohmann et al. 2012), but also for algal toxins (e.g., Li et al. 2011; Zendong et al. 2016). However, the polar organic chemical integrative sampler (POCIS) has been successfully applied to pharmaceuticals, estrogens, and polar pesticides down to the low ng/L range in marine waters (Martínez Bueno et al. 2016; Jones et al. 2019).

## Assessment of effects

Adverse effects of contaminant exposures can be assessed by different effect-based methods, such as in situ studies on field-collected organisms, caging studies in polluted sites as well as laboratory exposure studies using in vitro and in vivo bioassays. Advantages of in situ monitoring include assessment of effects reflecting true environmental conditions such as (life)long exposure to hazardous substances, bioavailability of pollutants, and the presence of chemical mixtures. A challenge is to link observed effect to a specific chemical. In contrast, in vitro and in vivo exposure studies are performed under highly controlled experimental settings, which increase their reproducibility. Despite low ecological relevance, many bioassays are considered valuable screening tools in risk assessment and a complement to field-based in situ monitoring or an entirely chemical monitoring approach (Wernersson et al. 2014, 2015; Carere et al. draft).

**In situ field monitoring** uses biomarkers to assess the toxicological effects of exposure to hazardous substances from the molecular, cellular, and physiological level to higher biological levels such as reproduction disorders and signs of disease in field-sampled organisms (reviewed by e.g., Lionetto et al. 2021). Biomarkers are considered as either “specific” or “general” depending on their specificity in response. Specific biomarkers respond to either certain substances, such as imposex induced by tributyltin, or a group of substances with similar mode of action, such as estrogenic substances or certain groups of pesticides, like imidacloprid inhibiting the acetylcholine esterase activity (AChE) or terbutryn, simazine, diuron, and atrazine inhibiting the photosystem II (PSII). In contrast, general biomarkers such as lysosomal membrane stability (LMS), liver histopathology, or fish disease index respond to several classes of chemicals, but can also respond to other environmental stressors. They often reflect a more general stress response such as genetic and cellular damage, histological alterations, and early life stage effects.

For the **in vitro bioassays**, a battery of mostly high throughput screening assays are established for the assessment of specific biological effects using cellular systems of reporter gene assays in addition to cellular response assays. These bioassays are tailor-made to assess effects such as various types of endocrine disruption, genotoxicity, cytotoxicity, oxidative stress or metabolic enzyme activation, and several other endpoints of relevance to the aquatic environment. In vitro bioassays that measure estrogenic effects would, thus, target substances such as EE2, E2, and E1, but also weaker estrogens such as nonylphenols. Correspondingly, in vitro bioassays measuring thyroid-transthyretin disruption (TTR-TRβ CALUX) would target several PFAS compounds, but also chlorinated paraffins (Sprengel et al. 2021).

By using short-term toxicity tests on whole organisms, **in vivo bioassays** are designed to assess effects such as algae growth, *Daphnia* immobilization or fish embryo vitality (Escher et al. 2014; Di Paolo et al. 2016; Neale et al. 2017) and are used to assess the response to exposure to a variety of chemicals.

The combination of passive sampling and effect-based analysis was reported by De Baat et al. (2019) who developed a surface water assessment strategy utilizing in situ field exposure tests as well as in vitro and in vivo bioassays to POCIS passive sampler extracts and by Moeris et al. (2021) who combined passive sampler seawater extracts with an in vivo bioassay using *Phaeodactylum tricornutum*. In a recent European inventory and assessment of more than 130 effect-based methods for toxic substances, the authors conclude that at least ten marine biomarkers can now be considered “mature” from a WFD and MSFD perspective and could be used to assess ecologically relevant effects of chemical mixtures (Carere et al. draft). Standard Operating Procedures, environmental assessment criteria as well as national monitoring programs exist. For others, the main issue that remains to be solved before use in a regulatory context is the availability of assessment criteria.

However, specific biomarkers and bioassays for the herein addressed compounds have so far primarily been developed and used for estrogens and estrogenic substances. Estrogenicity-specific biomarkers include the aberrant production of the egg yolk protein vitellogenin (VTG) and the presence of ovarian tissue in fish testis, so-called intersex. The in vitro bioassays ER-CALUX (Estrogen receptor-mediated, chemical-activated luciferase reporter gene expression) and YES (Yeast estrogen screen) are frequently applied to detect estrogenicity and Carere et al. (draft) conclude that such in vitro tests can now be considered mature enough to be considered in a WFD context, also presenting an approach on how to develop assessment criteria (“trigger values”). Hettwer et al. (2018) reported on the *Arxula*-yeast estrogen screen (A-YES) which is also applicable to saline water. However, for marine samples, sample enrichment is usually required, as achievable test detection limits are else often above the trigger value. Thus, YES testing in combination with SPE in surface water samples from the Baltic Sea was reported by Beck et al. (2006a) and Deich et al. (2020) (Table 3). However, both reported on the suppression of yeast growth, presumably as a result of high concentrations of estrogenically active compounds in the extracts and it was considered that those could also derive from the sample enrichment. Thus, less intense solid phase extraction in combination with a more sensitive in vitro bioassay such as the ER-CALUX might be an alternative to overcome generally very low marine concentrations.

## Combined techniques: Effect-directed analysis (EDA)

Effect-directed analysis (EDA) uses an integrated approach where bioassays are combined with sample fractionation to reduce sample complexity followed by chemical analysis (reviewed by Brack et al. 2016; Hong et al. 2016). This process is repeated until the chemicals responsible for the observed effect in a specific bioassay are identified. The EDA approach, therefore, aids in identifying the drivers of toxicity in the chemical mixtures often found in aquatic environments such as the Baltic Sea. The fractionation procedure in EDA uses different chromatography techniques separating chemicals according to their properties. Identification of toxic compounds in individual fractions is mainly performed using targeted, but also non-targeted or suspect MS methods (Hollender et al. 2019). For example, Brennan et al. (2020) proposed a strategy to design EDA for the analysis of endocrine-active compounds in water in combination with liquid chromatography (LC)-based fractionation and mass spectrometric analysis and Tufi et al. (2016) reported on the identification of AChE inhibitors in surface water through an EDA approach.

Despite an increasing use of EDA, relatively few studies have been performed on seawater extracts so far. However, Beck et al. (2006b) applied a fractionation technique based on reverse-phase chromatographic separation in combination with YES testing and chemical analysis to detect different types of estrogens and estrogenic mimicking compounds contributing to the estrogenic activity found in surface water samples from the German Baltic Sea coast. In addition, by using EDA, Booij et al. (2015) identified six herbicides in Dutch estuarine and coastal waters responsible for the inhibition of photosystem efficiency in marine microalgae. Finally, in an EDA approach, the estrogenic effect of the UV filter oxybenzone was identified (Schlenk et al. 2005; Zwart et al. 2018).

## Synthesis und recommendations for improved assessment of the state of the Baltic Sea

**Fig. 2:**
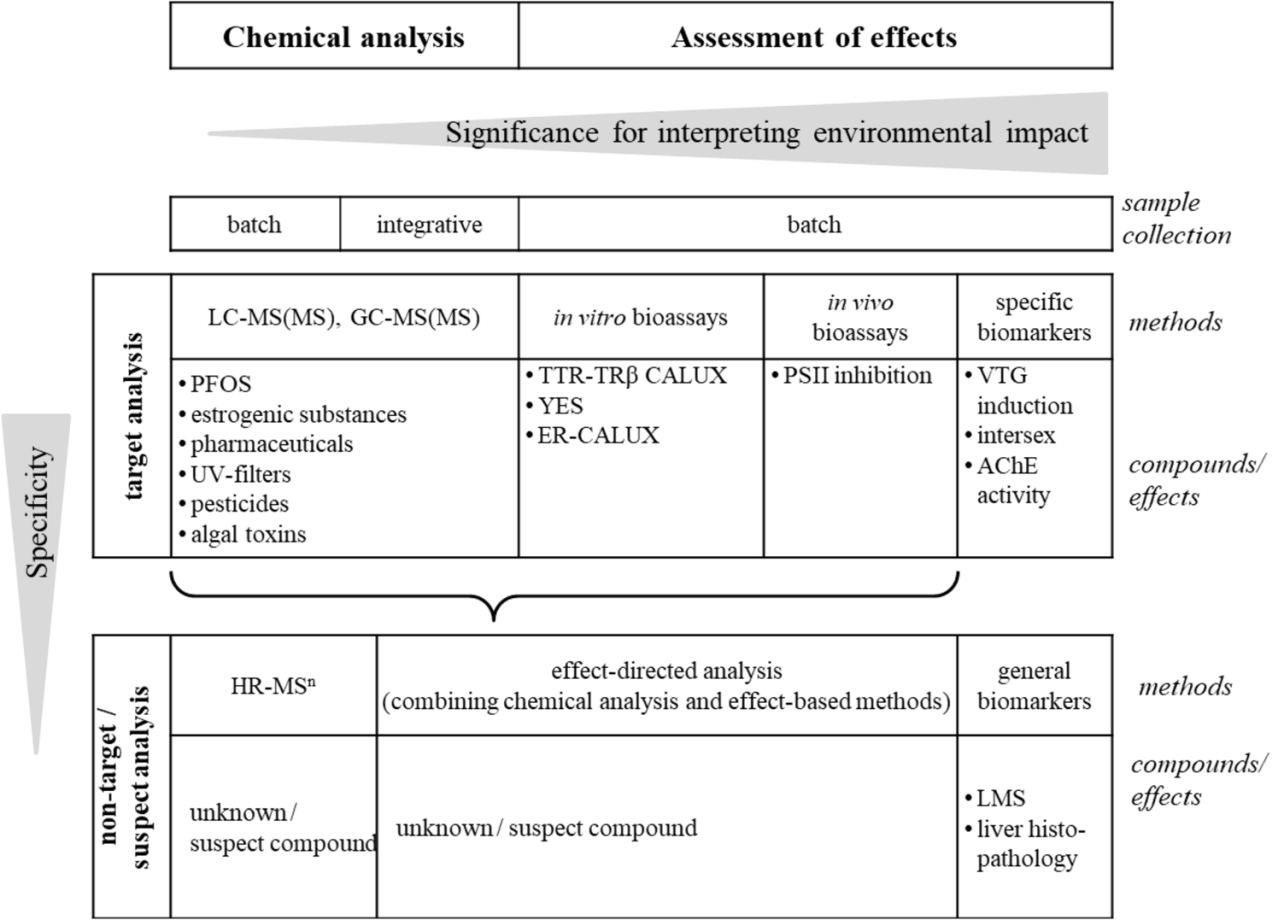
Overview and classification of example methods and compounds/effects utilized in chemical analysis as well as assessment of effects for the analysis of compounds of emerging concern in the Baltic Sea. Sample collection in “batch” corresponds to sampling at one time, while “integrative” is the sampling over a certain time period (passive sampling)

Negative effects on marine organisms to varying degree can result from the presence of the substances in focus of this review. There is a vast number of approaches to derive information on the presence of these compounds in the marine environment. They can be divided into methods of chemical analysis and those assessing an effect. State-of-the-art methods are presented herein; however, the different approaches provide diverse information with different significance for interpreting the impact of individual toxic chemicals and mixtures thereof on the marine environment (Fig. 2).

Contaminants of emerging concern are currently only partially addressed in the monitoring of the Baltic Sea. A broader involvement of the substances from the groups of pharmaceuticals, estrogenic compounds, UV filters, and polar pesticides in HELCOM programs, including the establishment of threshold values, will enable the consideration of their associated risks in the holistic assessment of the Baltic Sea. In addition to that, their chemical monitoring in seawater allows tracing the potential sources and paths into the Baltic Sea. Naturally produced algal toxins are currently not addressed in any Baltic Sea water policy context. Investigations on peak concentrations of these toxins would benefit from data collection on a weekly basis during a season with high biological activity. The spatiotemporal variations of the toxin concentrations render sessile, bottom dwelling filter feeders such as blue mussels suitable matrices for sampling for monitoring purposes. These organisms are especially exposed to algal toxins and other contaminants, as they accumulate high amounts of dissolved and particulate substances from the water column in combination with the fact that many filamentous algae are epiphytic on mussels and release chemical compounds during senescence and decay. Studies investigating concentrations and effects on higher organisms benefit from including a sum of the two groups MeO-PBDEs and OH-PBDEs.

Data on the free dissolved fraction of a compound obtained through passive (integrative) sampling could further substantiate determined seawater concentrations. This provides additional information on the compounds’ bioavailability and potential toxicity to organisms. Nonetheless, only effect-based methods provide a direct measure of current impact. Methods addressing effects that derive specifically from the herein addressed compounds are currently not implemented in HELCOM monitoring and are not mandatory in the WFD/MSFD context. The effect-based approach would fit very well into the HELCOM monitoring context, to cover substances not yet listed and the effects of mixtures of toxic substances. Assessment procedures and criteria are now available to cover at least the presence of estrogenic substances in surface waters and in vitro tests that measure thyroid disruption could be considered to at least partially cover some PFAS compounds.

Overall, chemical analysis alone will not provide sufficient information to assess the risks for marine organisms and, furthermore, cannot cover all substances. In this regard, it was shown for water samples from different sources, that the known effects of the therein detected chemicals could explain less than 0.1% of the observed induction of the oxidative stress response measured by an in vitro bioassay (Escher et al. 2013). Thus, it must be considered that unknown compounds present in the water largely contribute to toxic effects, which underlines the need for effect-based analysis for an efficient assessment. However, sole assessment of effects will also not provide enough information on the presence of individual substances or local and temporal trends of contaminant concentrations in the Baltic Sea. Thus, combining results from chemical analysis approaches with those from effect-based methods provide a clearer picture of the environmental risks that might derive from concentrations of contaminants and naturally produced toxins. For instance, a weight-of-evidence approach is widely used for the assessment of sediment sites. The sediment quality triad (Chapman 1990) integrates results from chemical analysis, toxicity testing, and in situ field monitoring to assess the potential risk that might derive from contaminated sediments. In addition, the International Council for the Exploration of the Sea (ICES) has produced detailed reports on integrated monitoring of contaminants and their effects (Davies and Vethaak 2012). This concept could be adapted towards seawater and would aid in determining the current state of the Baltic Sea.

## Conclusions

Pressure on Baltic Sea organisms arises from anthropogenically and naturally derived harmful substances. Negative effects to marine organisms can derive from substances of the compound groups pharmaceuticals, estrogenic compounds, UV filters, polar pesticides, and naturally produced algal toxins. Herein, we conducted literature searches, basically following the systematic literature review technique described by Mengist et al. (2020), on concentrations of substances of these compound classes in the Baltic Sea and on methodological advances for their chemical and effect-based analysis suitable for marine water. Our data review shows that individual substances of these compound groups are present in Baltic Sea water. However, they are currently hardly addressed in HELCOM or WFD/MSFD monitoring, meaning that associated risks are not taken into account in the holistic assessment of the state of the Baltic Sea.

The very low compound concentrations typical for marine waters, in combination with their chemical characteristics, require sensitive and robust methods and instrumentation for their analysis. Advances in the field of selective analyte enrichment from saline water as well as instrumental developments in the field of mass spectrometric techniques set the basis for the efficient, highly sensitive, and selective analysis in seawater. Apart from the mere chemical analysis, there is the need to include results from effect-based methods into the assessment to derive current impact of individual substances and compound mixtures on marine organisms. In this regard, a range of biomarkers and bioassays have already been developed for operational use in marine waters. The complementary use of different methodologies pointing towards different aspects of pressure might be a promising path for future contaminant and risk assessment in the Baltic Sea.

## Supplementary Information

Below is the link to the electronic supplementary material.

(PDF 120 kb)
