# Correction to: Substances of emerging concern in Baltic Sea water: Review on methodological advances for the environmental assessment and proposal for future monitoring

**DOI:** 10.1007/s13280-022-01731-1

**Published:** 2022-04-02

**Authors:** Marion Kanwischer, Noomi Asker, Ann-Sofie Wernersson, Marisa A. Wirth, Kathrin Fisch, Elin Dahlgren, Helena Osterholz, Friederike Habedank, Michael Naumann, Jaakko Mannio, Detlef E. Schulz-Bull

**Affiliations:** 1 Department of Marine Chemistry, Leibniz Institute for Baltic Sea Research Warnemünde, Seestraße 15, 18119 Rostock, Germany; 2 Department of Biological and Environmental Sciences, University of Gothenburg, Medicinaregatan 18A, 41390 Göteborg, Sweden; 3 Department for Management of Contaminated Sites, Swedish Geotechnical Institute, Hugo Grauers gata 5 B, 41296 Göteborg, Sweden; 4 Swedish University of Agricultural Sciences, Stångholmsvägen 2, 178 93 Drottningholm, Sweden; 5 State Office for Agriculture, Food Safety and Fisheries, Mecklenburg-Western Pomerania, Thierfelderstraße 18, 18059 Rostock, Germany; 6 Centre for Sustainable Consumption and Production/Contaminants, Finnish Environment Institute, Latokartanonkaari 11, 00790 Helsinki, Finland; 7 Department of Physical Oceanography and Instrumentation, Leibniz Institute for Baltic Sea Research Warnemünde, Seestraße 15, 18119 Rostock, Germany

## Correction to: Ambio 10.1007/s13280-021-01627-6

In the original published article the citation given as “Pleßow, A. 2007. *Organic pollutants in the water cycle properties, occurrence, analysis and environmental relevance of polar compounds*. *UWSF-Z Umweltchem Ökotox*, vol 19, ed. T. Reemtsma and M. Jekel. 10.1065/uwsf2007.03.181” does not reference the correct article.

The correct reference is provided below:

Heberer, T., and T. Ternes. 2006. Residues of Pharmaceuticals from Human Use. In *Organic pollutants in the water cycle*, eds. T. Reemtsma and M. Jekel, 41–63. Weinheim, FRG: Wiley–VCH Verlag GmbH & Co. KGaA. 10.1002/352760877X.ch2.

