# Supplementary material for: Substances of emerging concern in Baltic Sea water: Review on methodological advances for the environmental assessment and proposal for future monitoring
Source: Ambio. 2021 Oct 12;51(6):1588–608. doi: 10.1007/s13280-021-01627-6 (PMC9005613; doi:10.1007/s13280-021-01627-6)
Supplement: Supplementary file 1 — (PDF 120 kb) [file 13280_2021_1627_MOESM1_ESM.pdf]

Ambio

Electronic Supplementary Material

This supplementary material has not been peer reviewed.

Title: **Substances of Emerging Concern in Baltic Sea Water – Review on Methodological Advances for the Environmental Assessment and Proposal for Future Monitoring**

Authors: Marion Kanwischer, Noomi Asker, Ann-Sofie Wernersson, Marisa A. Wirth, Kathrin Fisch, Elin Dahlgren, Helena Osterholz, Friederike Habedank, Michael Naumann, Jaakko Mannio, Detlef E. Schulz-Bull

Table S 1 Conducted literature searches following the procedure described by Mengist et al. 2020. For this study, 3 review objectives were defined and the specifications of the conducted searches are described in the respective cell columns. The literature searches were performed from April 2020 to July 2021.

<table>
<tr><td colspan="3">REVIEW OBJECTIVES AND SCOPE OF THE SEARCHES</td></tr>
<tr><td>Which concentrations for contaminants of emerging concern are reported for Baltic Sea surface water?</td><td>Which methodological advances are reported for the chemical analysis of contaminants of emerging concern in marine water?</td><td>Which effect-based methods exist that could be used for marine water analysis?</td></tr>
<tr><td></td><td></td><td></td></tr>
<tr><td colspan="3">TARGET COMPOUND CLASSES</td></tr>
<tr><td colspan="3">pharmaceuticals / UV-filters / estrogens and estrogenic compounds / polar pesticides / per- and polyfluoroalkyl substances / algal toxins</td></tr>
<tr><td colspan="3">ADDITIONAL CLASSIFICATION</td></tr>
<tr><td>chemical analysis / effect-based analysis</td><td>sample processing / analytical approaches / passive sampling</td><td>in situ field monitoring / in vitro bioassays / effect-directed analysis</td></tr>
<tr><td colspan="3">SEARCH STRATEGY, SOURCES</td></tr>
<tr><td>• databases Web of Science and HELCOM</td><td colspan="2">• database Web of Science or Science Direct
• reports of marine science organizations
• reports of water policy working groups</td></tr>
<tr><td colspan="3">SEARCH KEYWORDS</td></tr>
<tr><td>pharmaceuticals | UV-filter | estrogen | pesticide | perfluoroalkyl | PFAS | nodularin | microcystin | polybrominated phenols | polybrominated dibenzo-p-dioxins | AND Baltic Sea</td><td colspan="2">• sample processing:
solid-phase extraction | AND pharmaceutical | UV-filters | UV-stabilizers | estrogen | pesticide | PFAS | perfluoroalkyl |algal toxins AND seawater | marine
• analytical approaches:
HRMS | FT-ICR-MS | Orbitrap | MS-MS mass-spectrometry | multi method AND contaminant AND marine | ocean | seawater
• passive sampling:
passive sampling AND seawater
• effect-based methods:
effect-based | in vitro bioassay | in vivo bioassay | effect-directed analysis AND marine | seawater | water</td></tr>
<tr><td colspan="3">INCLUSION CRITERIA</td></tr>
<tr><td>• within review objective from title and abstract
• accessible
• English language</td><td colspan="2">• within review objective from title and abstract
• accessible
• English language
• reports not older than 2010
• method applied in seawater or promising for seawater analysis</td></tr>
<tr><td colspan="3">EXTRACTED INFORMATION</td></tr>
<tr><td>• Baltic Sea study area
• study year
• mode of assessment (chemical or effect-based)
• method details
• surface seawater conc.</td><td colspan="2">• description of the method
• reported limitations
• example study sites and key findings</td></tr>
<tr><td colspan="3">ANALYSIS</td></tr>
<tr><td colspan="3">• summary or reported data
• outlining predominant substances within compound classes for the Baltic Sea
• describing methods for chemical and effect-based analysis potentially suitable for analysis of marine water
• built recommendations for improved assessment of the state of the Baltic Sea on data</td></tr>
</table>

References

Mengist, W., T. Soromessa, and G. Legese. 2020. Method for conducting systematic literature review and meta-analysis for environmental science research. *MethodsX* 7: 100777. doi:10.1016/j.mex.2019.100777.
